# Development of Improved Confined Compression Testing Setups for Use in Stress Relaxation Testing of Viscoelastic Biomaterials

**DOI:** 10.3390/gels10050329

**Published:** 2024-05-13

**Authors:** Anthony El Kommos, Alicia R. Jackson, Fotios Andreopoulos, Francesco Travascio

**Affiliations:** 1 Department of Biomedical Engineering, University of Miami, Coral Gables, FL 33146, USA; axe627@miami.edu (A.E.K.); a.jackson2@miami.edu (A.R.J.); 2 Department of Mechanical and Aerospace Engineering, University of Miami, Coral Gables, FL 33146, USA; 3 Department of Orthopaedic Surgery, University of Miami, Miami, FL 33136, USA; 4 Max Biedermann Institute for Biomechanics, Mount Sinai Medical Center, Miami Beach, FL 33140, USA

**Keywords:** hydrogel, equilibrium force, aggregate modulus, peak force, confined compression

## Abstract

The development of cell-based biomaterial alternatives holds significant promise in tissue engineering applications, but it requires accurate mechanical assessment. Herein, we present the development of a novel 3D-printed confined compression apparatus, fabricated using clear resin, designed to cater to the unique demands of biomaterial developers. Our objective was to enhance the precision of force measurements and improve sample visibility during compression testing. We compared the performance of our innovative 3D-printed confined compression setup to a conventional setup by performing stress relaxation testing on hydrogels with variable degrees of crosslinking. We assessed equilibrium force, aggregate modulus, and peak force. This study demonstrates that our revised setup can capture a larger range of force values while simultaneously improving accuracy. We were able to detect significant differences in force and aggregate modulus measurements of hydrogels with variable degrees of crosslinking using our revised setup, whereas these were indistinguishable with the convectional apparatus. Further, by incorporating a clear resin in the fabrication of the compression chamber, we improved sample visibility, thus enabling real-time monitoring and informed assessment of biomaterial behavior under compressive testing.

## 1. Introduction

The design of cell-based biomaterial alternatives shows great promise in the field of tissue engineering. There is a need for accurate recapitulation of the functional properties of the tissues in order to improve the efficacy of the substitute biomaterials. Cartilaginous tissues, such as the articular cartilage and meniscus found in the knee, are complex, multilayered, highly organized tissues responsible for absorbing high dynamic loads and dissipating stress within the knee joint [1,2]. Healthy cartilage plays a vital role in maintaining joint health, preventing injury, and preventing long-term joint degeneration [1,3,4,5].

In order to produce tissue substitutes, researchers must be able to accurately characterize native tissue, as well as any potential biomaterial substitutes, according to guidelines set forth by the Food and Drug Administration (FDA) and International Cartilage Repair Society (ICRS) [6]. Most notably, in the field of cartilaginous tissue engineering, the aggregate modulus (H_A_) is evaluated by means of compression testing. The aggregate modulus is a time-dependent measure of the ability of the native tissue or a biomaterial to withstand compressive stresses via internal fluid pressurization during compression (elastic), followed by relaxation of internal stresses within the polymer network by means of fluid exudation (viscous) under constant strain [6,7,8,9,10]. Those measurements are important in the development of biomaterials as cartilage tissue substitutes that need to replicate the complex time-dependent poroelastic stress relaxation behavior of the native tissue [1]. However, no universal guidelines currently exist as to preferred testing protocols for biomaterial replacements.

**Figure 1:**
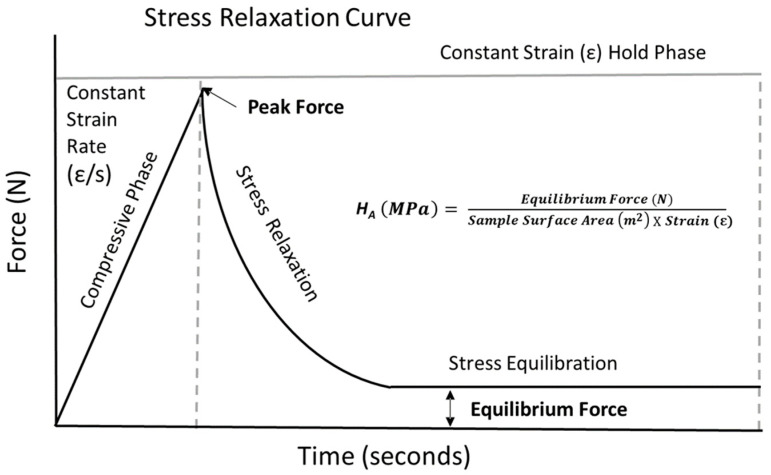
Sample stress relaxation curve highlighting peak force extracted following compressing region, along with equilibrium force value extracted following extended hold phase stress relaxation period. Equilibrium force values are then extracted and utilized to calculate the aggregate modulus (H_A_) utilizing the equation depicted above.

To assess poroelastic behavior in biomaterial characterization, stress relaxation compressive testing is a common method: the material’s stress response is monitored over time during a constant strain rate compressive phase, followed by an extended hold phase at a constant strain [7,11], allowing for the calculation of relevant mechanical properties [8]. Previous studies show that numerous factors such as strain rate, sample size, biomaterial composition, and apparatus design can have significant effects on testing results [6]. Importantly, in order to accurately assess the mechanical performance of a biomaterial substitute, the testing system must have a large enough range to capture the peak forces generated during the compressive phase, while also maintaining accuracy at low forces during the equilibration period of the hold phase [12]. In turn, using a load cell of appropriate accuracy relative to biomaterial composition and surface area is important for assessing the peak forces, equilibrium force, and aggregate modulus (H_A_) (Figure 1).

**Figure 2:**
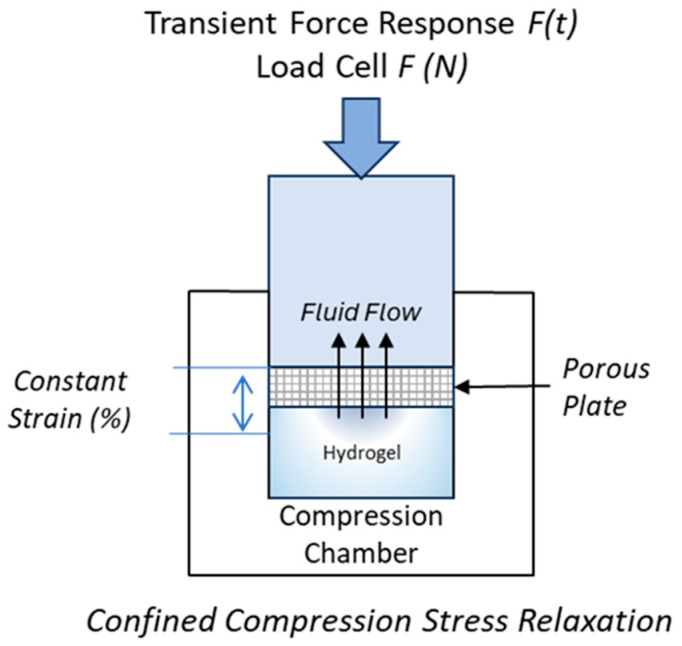
Diagram depicting confined compression stress relaxation of hydrogel biomaterial. A porous plate is affixed to a compressing plug, compressed to a constant strain rate, and held for a period as the transient stress response is measured.

Stress relaxation testing of biomaterials and native tissue is performed either in unconfined or confined configurations. According to a literature review by Patel et al., the average sample diameter tested in confined compression is 4.85 ± 1.25 mm [6]. Sample diameters in the literature range from 3–10 mm, based on sample type: bovine, human, porcine, or scaffold [1,6,7,10,12]. One typical confined compression setup is composed of an impermeable compression chamber for the sample, along with a corresponding compressing plug and affixed porous plate allowing for fluid exudation (Figure 2) [6,7,8,13,14]. While tissue dimensions may limit sample size production, a small sample surface area provides challenges in accurately assessing the biomechanics of biomaterials.

The combination of small sample surface area coupled with low strain rates generates low force responses during compressive testing. While utilizing a more sensitive load cell could allow for improved accuracy at lower force values, the total force range required to thoroughly test the poroelastic behavior of biomaterial substitutes would be compromised. As a result, increasing the sample surface area allows for testing of the complete range of forces, while simultaneously increasing the force response at lower strain rates.

In addition to sample size considerations, given the anisotropic and complex structure of human tissues such as articular cartilage or the fibrocartilginous meniscus, visibility during testing can aid in biomaterial characterization [15,16]. Stereolithography Additive (SLA) manufacturing technology allows for the development of complex geometries by utilizing light-based photopolymerizable resins with a variety of properties, including optical transparency and mechanical resiliency, thus allowing for the development of various testing setups in multiple orientations and sizes.

Here, we present a novel confined compression chamber developed to aid in the characterization of biomaterials for tissue engineering. Our primary objectives were to improve accuracy and sample visibility. By increasing the sample surface area, we aim to improve accuracy at lower strain rates and strains while simultaneously maintaining the overall force range for thorough characterization of viscoelastic behavior. Furthermore, we highlight the use of clear resins via SLA that allows the user to visually confirm sample placement in test setup, improving consistency and repeatability. To validate our new testing system, several hydrogel compositions were assessed on both the conventional and revised confined compression testing setups and the resulting equilibrium forces, aggregate moduli, and peak force values were analyzed.

## 2. Results and Discussion

The goal of this study was to develop and validate an optimized system for the assessment of biomaterials that allows for the capture of the appropriate range of force values, improved accuracy, and sample visualization during testing. This novel confined compression setup aids in biomaterial characterization by increasing the resultant force values, maintaining load cell range, and improving testing setup and consistency, resulting in improved data accuracy during the evaluation of the poroelastic behavior of biomaterials. Moreover, the described manufacturing and design processes can be further adapted for the compressive testing of various hydrogels and tissues at a low fabrication cost and short manufacturing time.

### 2.1. Changes in the Compression Testing Setup

**Figure 3:**
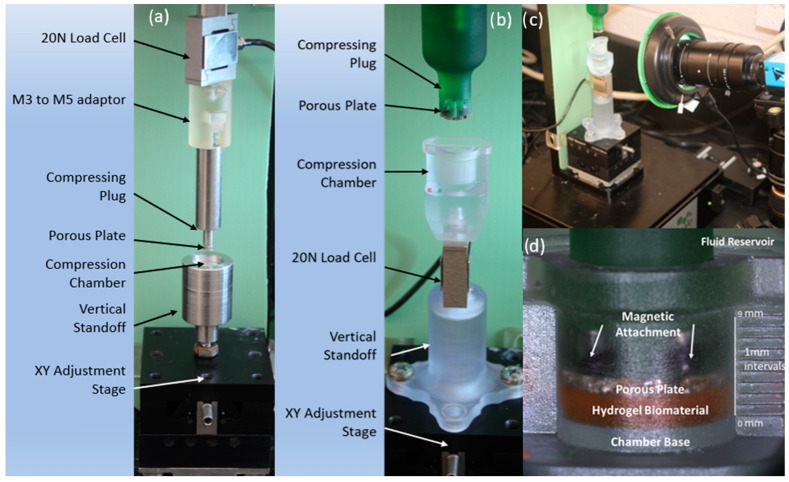
(**a**) Conventional confined compression setup utilizing a 20 N load cell attached to the top linear actuator, compressing plug and compression chamber attached to an XY adjustment stage. (**b**) Revised confined compression setup utilizing a vertically oriented load cell below the compression chamber attached to an adjustable XY stage and compressing plug attached to the vertical actuator. (**c**) Optical transparency of the SLA printed material allowed for the addition of a macro camera to aid in setup and testing. (**d**) Macro image of the revised compression chamber.

The initial confined compression setup consisted of a 20 N (0.2% accuracy rating ±0.04 N) load cell vertically oriented on the top linear actuating portion of a UniVert Uniaxial testing machine (CellScale, Waterloo, ON, Canada). A metal compressive plug was attached to the bottom of the load cell with a fine porous plate (5 mm diameter) affixed to the bottom via glue. The steel sample chamber was attached to an adjustable XY stage for adjustment of the chamber (Figure 3a). The compressive chamber dimensions were a 5 mm diameter and a 4 mm depth. Samples were prepared using a 5 mm diameter trephine punch yielding a sample surface area of 19.63 mm^2^.

The revised setup was designed with the intention of mitigating sources of error encountered in our conventional setup by increasing sample surface area and utilizing optically transparent SLA printing to allow for imaging with a macro-image camera (DFK 33UX264) during setup and testing for improved accuracy (Figure 3c). The novel confined compression testing setup was designed using CAD software (Autodesk Fusion360) and fabricated using SLA manufacturing (Figure 3b). The load cell position was re-oriented from the top linear actuating portion of the setup to below the compression chamber, allowing for axial loading through the sample chamber while remaining in a static position (Figure 3a,b).

The sample chamber diameter was increased from 5 mm (19.63 mm^2^ top surface area) to 12.7 mm (1/2 inch, 126.7 mm^2^ top surface area), resulting in a sample surface area increase of 645%. The surface area increase was made to produce a stronger force response, primarily during the equilibration period at low strain rates, and to provide a more heterogenous biomaterial sample due to a larger volume. Further, by increasing the sample diameter, the initial compressive peak forces increased towards the desired load cell midrange values (~20–80% max rating). The 12.7 mm diameter was chosen for punch availability and compatibility with available porous plate diameters.

The porous plate attachment to the compressing plug was modified to allow for magnetic attachment. This was accomplished by embedding magnets into the compressing plug and sealing with resin to prevent fluid contact and rust development (Figure 3c,d). The magnetic suspension allows the porous plates to shift slightly in the XY plane, allowing for self-centering during compression, ultimately reducing friction during the compressive phase of testing and preventing false readings [6].

**Figure 4:**
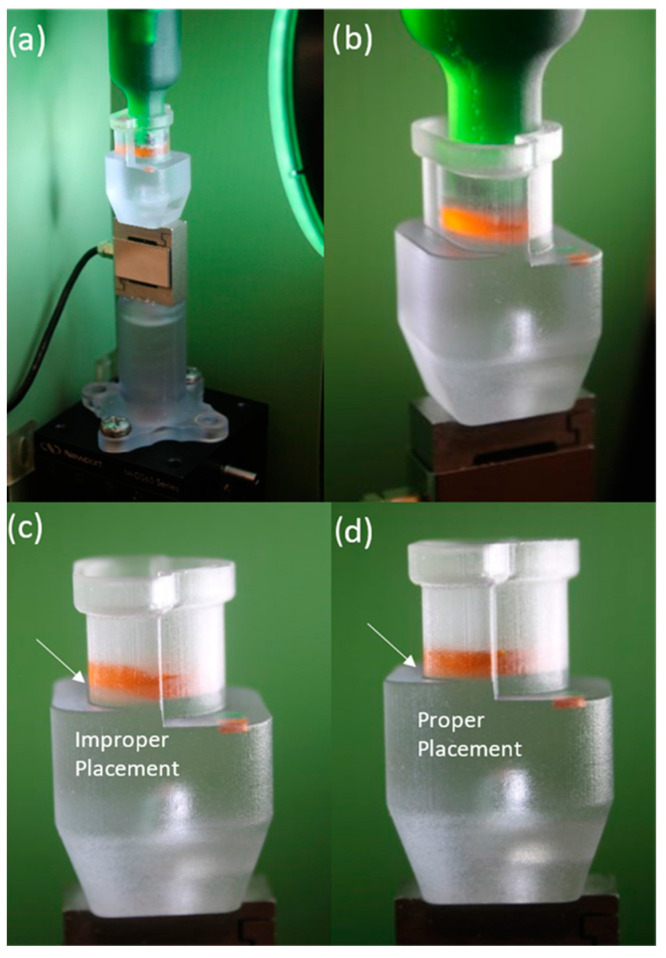
(**a**) Macroscopic view of revised setup highlighting visibility of sample within chamber. (**b**) Close up image of compressing plug lowered to contact hydrogel showing 360-degree sample visibility when seated in chamber. (**c**) Close up image of improper sample placement showing spacing between the bottom surface and hydrogel. (**d**) Proper sample placement showing hydrogel placement even across chamber bottom surface. The hydrogel sample was dyed for visualization.

Macro images were used to validate proper sample placement within the chamber, alignment of the compressing plug and chamber, and the contact surface of the porous plate and hydrogel sample (Figure 3d). Additionally, optical transparency allowed for visualization of the sample within the compression chamber, to ensure the sample was properly seated in the chamber base (Figure 4).

### 2.2. Comparison of Results for Conventional and Revised Setups

A summary of equilibrium forces, aggregate moduli, and peak forces for the gelatin and agarose hydrogels utilizing the conventional and revised setups is provided in Table 1.

**Figure 5:**
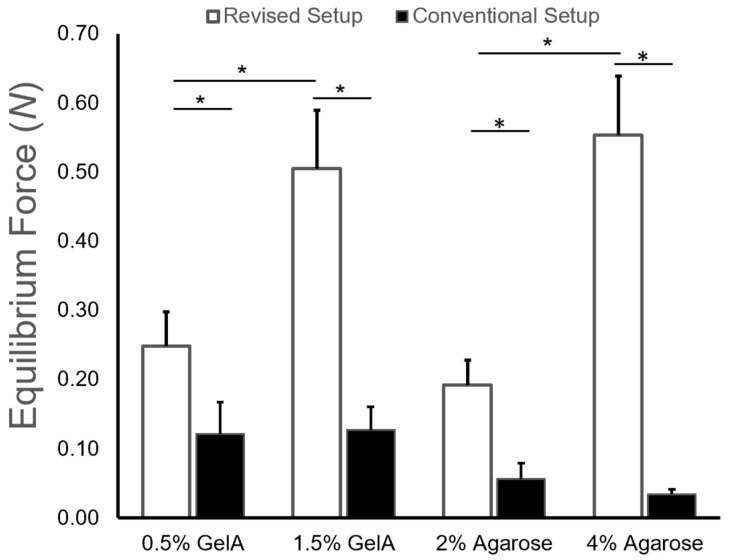
Equilibrium force values obtained for both cross-linking conditions on initial setup and revised setup at a strain rate of 10%, (*) indicating statistical significance (*p* < 0.05) across either cross-linking conditions or setup.

For the equilibrium forces measured (Figure 5), statistical analyses showed significant effects for both the gel type (*p* < 0.001) and setup (*p* < 0.001), as well as a significant interaction between the factors (*p* < 0.001). For the GelA hydrogels, there were significant differences between the equilibrium force measurement for each gel type using different testing setups (0.5%: *p* = 0.0013; 1.5%: *p* < 0.001). The same was also true when comparing measurements for the agarose gels between the two setups (2%: *p* < 0.001; 4%: *p* < 0.001). When comparing the values for different hydrogel groups, only the revised setup detected significant differences between hydrogel preparation (GelA: *p* < 0.001; agarose: *p* < 0.001). In contrast, there were no significant differences between hydrogel formulations for the conventional setup (GelA: *p* = 0.878; agarose: *p* = 0.548), due to poor load cell resolution at the resulting equilibrium force values.

The conventional setup utilized a smaller sample surface area (5 mm dia.) which, during hydrogel compression testing (at 10% strain), produced a low equilibrium force response relative to the 20 N load cell used. As a result, the conventional setup was not able to detect statistically significant differences between the equilibrium force values for different gelatin or agarose hydrogel formulations. This is likely the result of the low force responses generated by the small surface area of the hydrogel samples, biomaterial compressive stiffness, and load cell accuracy (0.2% accuracy rating ±0.04 N).

To improve the accuracy of the testing system, in the revised setup, the sample diameter was increased from 5 mm to 12.7 mm, correlating to a sample surface area increase of 645%. Average equilibrium force values for the 0.5% and 1.5% GelA hydrogel concentrations increased 205% and 399%, respectively, while aggregate modulus values fell to 34% and 49%, respectively, relative to the conventional setup (Figure 5). The revised setup resulted in statistically significant differences in equilibrium force values between gelatin hydrogels with different crosslinking densities. Similarly, the average equilibrium force values for the 2% and 4% agarose hydrogel concentrations increased 341% and 1640%, respectively (Figure 5). Moreover, the revised setup also yielded statistically significant differences in equilibrium force data between agarose gel concentrations.

Given that the primary metric in stress relaxation testing is the load cell force data, the increased equilibrium force produced for all hydrogel concentrations tested in the revised setup improves data accuracy, while maintaining load cell force range. In addition, the increase in sample surface area and volume allows for a more heterogenous depiction of hydrogel formulation as any localized cross-linking density, stress concentrations, or sample imperfections from production processes are averaged over a larger area during testing [17].

**Figure 6:**
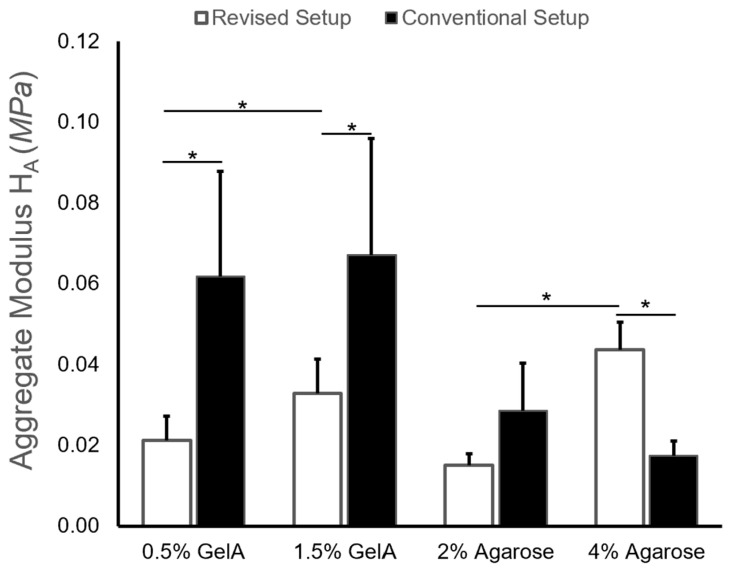
Aggregate modulus H_A_ values obtained for both cross-linking conditions on initial setup and revised setup at a strain rate of 10%, (*) indicating statistical significance (*p* < 0.05) across either cross-linking conditions or setup.

Aggregate modulus values determined by the two setups followed similar trends to those for the equilibrium force measurements (Figure 6). Statistical analyses showed significant effects of both the gel type (*p* < 0.001) and setup (*p* < 0.001), as well as a significant interaction between the factors (*p* < 0.001), on H_A_. When comparing the H_A_ of each gel type measured by different setups, significant differences were found for 0.5% GelA (*p* = 0.0011), 1.5% GelA (*p* = 0.0076), and 4% agarose (*p* = 0.0099), while no significant difference was observed for the 2% agarose gels (*p* = 0.170). Similar to the equilibrium force measurements, significant differences between the H_A_ of different gel formulation groups were only found for the revised setup (GelA: *p* = 0.0422; agarose: *p* = 0.0054), but not for the conventional setup (GelA: *p* = 0.576; agarose: *p* = 0.252). Again, this is likely due to the low forces generated by the small surface area of hydrogels, biomaterial compressive stiffness, and load cell accuracy on the conventional setup. Aggregate modulus value fell to 53% of the initial setup for the 2% agarose gel, and increased 250% relative to the conventional setup for the 4% agarose hydrogel (Figure 6).

Agarose hydrogels are a common hydrogel biomaterial used for a variety of applications in tissue engineering, and are often utilized as a control group in the mechanical characterization of biomaterials [6]. The revised setup yielded aggregate modulus values of 15 kPa and 44 kPa for the 2% and 4% agarose hydrogels, respectively, with literature values ranging from ~2–40 kPa for similar agarose concentrations [13,18,19,20]. Moreover, the revised setup also exhibited trends consistent with literature as aggregate moduli and equilibrium forces increased with polymer concentration and cross-linking density for both hydrogels [21,22].

### 2.3. Discussion of Findings

In biomaterial characterization, capturing a dynamic range of force values during stress relaxation testing allows for the calculation of important properties, such as elastic modulus, relaxation rates, relaxation time, and time constant [23]. The previous literature has investigated the effect of ideal vs. non ideal testing conditions in confined compression, and estimates the relative error to be low for aggregate modulus (~2%), but significant for other metrics such as hydraulic permeability (~30%) [11]. Therefore, capturing accurate data across a physiologically relevant range is vital for the proper biomechanical evaluation of tissue substitutes.

For all of our testing, samples were compressed at a rate of 3.12 µm/s or 0.1%/s to a strain of 10%. A recent review by Patel et al. showed that 0.1%/s was the lowest normalized rate typically utilized in compressive ramp testing [6]. Given the effect of strain rate on compressive testing, we used the lowest normalized strain rate in order to validate our setup, as increased strain rates generate increased force responses during compressive testing [6,12].

In our measurements, peak force values were extracted from the compressive region, and equilibrium force values were generated from the stress–strain curve across the ideal operating range and accuracy of the 20 N load cell that was used in our apparatus. The revised setup retains the ability to capture peak force values while improving the accuracy needed at lower equilibrium forces (Table 1). Moreover, for the revised setup, the peak force values increased as a function of hydrogel crosslinking consistent with previous literature [17,21].

The modifications that were made in the revised setup improve testing conditions and help to mitigate human error. The addition of a magnetic plate suspension allowed for a slight shift of the porous plate within the compression chamber, reducing friction during compression that could potentially alter recorded force values. Moreover, porous plates of varied pore sizes can be readily interchanged providing an additional parameter to help characterize biomaterial behavior. The clear resin used to fabricate the compressive chamber allowed for visualization of the hydrogel sample within the chamber, ensuring proper sample placement. By observing the initial contact point between the porous plate and hydrogel sample, we can further validate testing consistency by ensuring samples are properly preloaded prior to medium introduction. This added functionality could prove useful when assessing composites, multi-layered biomaterials, or non-uniform deformation in biomaterials.

## 3. Conclusions

In this study, we report the development of a simple yet novel testing apparatus for the mechanical assessment of hydrogel samples in confined compression. Using additive manufacturing techniques, we were able to fabricate a customizable and cost-effective compression chamber that could be used to assess the mechanical properties of various biomaterials and tissue samples with variable sample sizes. The revised setup also allows for visual confirmation of proper sample placement within the confined compression chamber and visualization of the porous plate interface with the hydrogel, thereby improving testing conditions and data accuracy. Potential limitations of this system include the porous plate availability and its sizing, due to the difficulty of printing plates with micrometer pore sizes. Furthermore, for the mechanical evaluation of softer biomaterials a larger confined compression chamber would need to be utilized, and a smaller load cell would need to be substituted. The presented setup has been optimized for the measurement of hydrogels in the range of those presented here; future studies will make additional improvements to overcome the limitations noted above and allow for assessment of a larger range of materials.

In summary, we have presented a novel testing setup for the mechanical assessment of hydrogel samples in confined compression, and showed that this setup can accurately measure mechanical properties of several hydrogel formulations. The revised setup also allows for visual confirmation of proper sample placement and porous plate interface with the hydrogel, thereby improving on testing setup and consistency compared to conventional setups. Further, the versatility of additive manufacturing allows production of a variety of future testing setups to aid in development and testing of either native tissue or biomaterials. Given that additive manufacturing is not limited relative to geometry, any shape and orientation of tissue or biomaterial can be tested. Allowing for a customizable cost-effective design process that can expedite research and further the field of biomaterial development for tissue engineering.

## 4. Materials and Method

### 4.1. Hydrogel Preparation

Gelatin hydrogels were prepared by first dissolving 3 g of GelA (Gelatin Type A from Porcine skin, 300 bloom, Sigma-Aldrich, St. Louis, MO, USA) in 20 mL of PBS to produce gelatin solutions at 0.15 g/mL concentrations. The gelatin solution was heated in a water bath to 65 °C to achieve complete solubilization. Glutaraldehyde stock solution (25% stock solution, Sigma-Aldrich) was diluted in phosphate-buffered saline (PBS) solution to concentrations of 0.5% and 1.5% vol/vol, and then it was immediately added and stirred to homogeneity with the heated GelA solution in a 10:1 volume to volume ratio (3ml GelA: 0.3 mL Glutaraldehyde). The solution was poured into a 6 well plate with inner diameter of 35 mm to produce gels with 3 mm thickness. The gelatin-glutaraldehyde solution was allowed to crosslink for one hour. The produced hydrogels were then incubated in PBS and swollen to osmotic equilibrium for 12 h at 4 °C.

Agarose solutions were prepared in concentrations of 2% and 4% wt./vol. by dissolving agarose (Invitrogen UltraPure Agarose 100, Thermo Fisher Scientific, Waltham, MA, USA) in PBS. The solutions were heated to 75 °C until complete dissolution, and a 3 mL sample was poured into a 6 well plate. Hydrogels were allowed to set for one hour, followed by incubation in PBS to swell to osmotic equilibrium for 12 h at 4 °C.

### 4.2. 3D Printing and Material Selection

**Figure 7:**
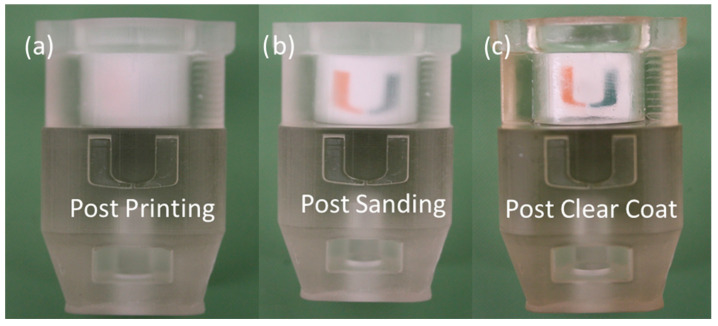
Progressive transparency improvement of additively manufactured parts via post- processing. (**a**) Initial clarity following printing was improved by using (**b**) wet sanding using progressively finer grit sandpaper (800, 1000, 3000, 5000) on the outer surfaces to maintain dimensional accuracy. (**c**) A thin layer of matte clear coat was then applied to the outer surfaces.

The revised compression chamber and compressing plug were printed using a Carbon3D M1 DLS SLA (digital light synthesis stereolithography) 3D printer (Carbon, Inc., Redwood City, CA, USA) at 50 µm Z-layer height with Loctite Henkel 405 Clear Resin (Young’s Modulus~1350 MPa). Printed parts were oriented for optimal part quality, including optical transparency of the compressive chamber, and dimensional accuracy within the XY plane for features requiring accuracy in tolerancing. Optical clarity of 3D printed parts was improved via post-processing with wet sanding, polishing, and clear-coat application (Figure 7).

Static stress deformation analysis was performed in Fusion 360 (Autodesk, Inc., San Francisco, CA, USA) to validate material selection. A 20 N load (Load Cell upper limit) was applied axially to the bottom surface of the compressive chamber to assess material displacement. Maximum Z deformation was determined to be negligible at 1.4 µm under 20 N static load.

### 4.3. Confined Compression Stress Relaxation Procedure

Prior to testing, the load cell was calibrated according to ASTM E4 and ISO-7500-1 standards [24]. Hydrogel samples, prepared as described below, were punched using the appropriate diameter sample punch (i.e., 5 mm or 12.7 mm) immediately upon removal from refrigeration. Prior to testing, samples were preloaded with 0.15 N and compressed for 1 min to ensure proper contact between the hydrogel and porous plate, prior to introduction of room temperature (22 °C) phosphate-buffered saline (PBS) solution into the testing well.

The UniVert CellScale’s linear actuator moves in micro step increments of 1.56 µm. A rate of 3.12 µm/s (2 micro-steps/s) was used to ensure consistency in the compression rate across samples. Samples were compressed at a rate of 3.12 µm/s (0.1%/s normalized) to a strain of 10% based on sample thickness, then held at constant strain for 1 h. Data was then processed using a custom Python script to assess peak force, equilibrium force, and aggregate modulus.

### 4.4. Revised Setup Procedure

**Figure 8:**
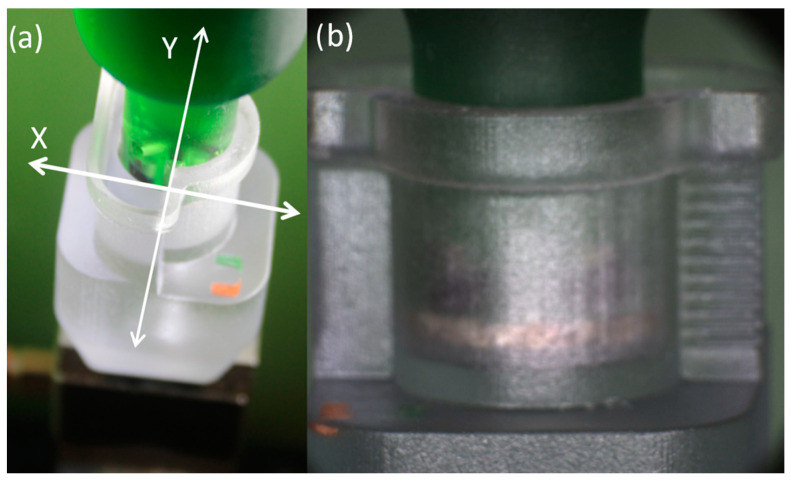
(**a**) Image of plug lowered within compression chamber as XY plane adjustment is performed utilizing the adjustment stage while monitoring force response and (**b**) macro camera image to ensure magnetic plate attachment.

To reduce friction during testing, the compressing plug was aligned via XY stage adjustment by utilizing the macro camera image and force response as the plug was manually jogged between the top and bottom surface of the chamber. Plug and chamber alignment was validated by moving the plug vertically within the chamber while generating no force response and maintaining magnetic attachment with the porous plate (Figure 8).

Imaging was then utilized to visualize gel position within the chamber and initial contact between the porous plate and hydrogel top surface during test setup (Figure 3d). Proper contact between hydrogel and porous plate was determined by force response and visual fluid exudation at the hydrogel porous plate interface. PBS was then introduced into the testing chamber.

### 4.5. Statistical Analysis

To assess the accuracy of the revised setup, a two-way ANOVA was conducted to investigate significant interactions and main effects on the equilibrium force and aggregate modulus values with factors being the gel type (0.5% GelA, 1.5% GelA, 2% agarose, 4% agarose) and testing setup (conventional or revised). Subsequently, Fisher’s LSD post hoc tests were then performed to assess the differences in means across the relevant comparisons. Statistical analyses were conducted using Minitab^®^21.4 statistical software (Minitab, LLC, State College, PA, USA). A 95% confidence interval, or significance level of (α = 0.05), was used for all tests. Results reported are mean ± standard deviation.

## Figures and Tables

**Table 1:** Summary of values for aggregate modulus, equilibrium force and peak force for both the conventional setup and revised setup. Values are reported as Mean ± Std. Dev.

	Gel Type	Conventional Setup	Revised Setup
Equilibrium Force (N)	0.5% GelA	0.121 ± 0.046	0.248 ± 0.050
	1.5% GelA	0.127 ± 0.033	0.505 ± 0.085
	2% Agarose	0.056 ± 0.023	0.192 ± 0.036
	4% Agarose	0.034 ± 0.007	0.554 ± 0.085
Aggregate Modulus (MPa)	0.5% GelA	0.062 ± 0.026	0.021 ± 0.006
	1.5% GelA	0.067 ± 0.029	0.033 ± 0.009
	2% Agarose	0.029 ± 0.012	0.015 ± 0.003
	4% Agarose	0.017 ± 0.004	0.044 ± 0.007
Peak Force (N)	0.5% GelA	1.077 ± 0.383	0.430 ± 0.162
	1.5% GelA	0.348 ± 0.054	1.489 ± 0.255
	2% Agarose	0.144 ± 0.059	0.327 ± 0.067
	4% Agarose	0.101 ± 0.016	1.113 ± 0.229

## Data Availability

The raw data supporting the conclusions of this article will be made available by the authors on request.
